# Prescribing cascades: do we need this construct? A reply

**DOI:** 10.1007/s41999-025-01315-8

**Published:** 2025-10-08

**Authors:** Paula A. Rochon, Denis O’Mahony, Antonio Cherubini, Graziano Onder, Mirko Petrovic, Kieran Dalton, Lisa M. McCarthy, Shelley A. Sternberg, Donna R. Zwas, Nathan M. Stall, Christina E. Reppas-Rindlisbacher, Nathalie van der Velde, Sarah N. Hilmer, Wei Wu, Joyce Li, Amy Ly, Jerry H. Gurwitz

**Affiliations:** 1 Women’s Age Lab and Women’s College Research Institute, Women’s College Hospital, Toronto, ON Canada; 2 ICES, Toronto, ON Canada; 3 Department of Medicine, University of Toronto, Toronto, ON Canada; 4 Institute of Health Policy, Management & Evaluation, Dalla Lana School of Public Health, University of Toronto, Toronto, ON Canada; 5 Sinai Health System, Toronto, ON Canada; 6 Department of Medicine (Geriatrics), University College Cork, Cork, Ireland; 7 Department of Clinical and Molecular Sciences, Università Politecnica Delle Marche, Ancona, Italy; 8 Geriatria, Accettazione Geriatrica e Centro Di Ricerca Per L’invecchiamento, IRCCS INRCA, Ancona, Italy; 9 Department of Geriatrics, Orthopedics and Rheumatology, Università Cattolica del Sacro Cuore, Rome, Italy; 10 Center of Aging, Fondazione Policlinico Universitario Gemelli IRCCS, Rome, Italy; 11 Department of Internal Medicine and Paediatrics, Ghent University, Ghent, Belgium; 12 Pharmaceutical Care Research Group, School of Pharmacy, University College Cork, Cork, Ireland; 13 Leslie Dan Faculty of Pharmacy, University of Toronto, Toronto, ON Canada; 14 Institute for Better Health, Trillium Health Partners, Toronto, ON Canada; 15 Women’s College Research Institute, Women’s College Hospital, Toronto, ON Canada; 16 Division of Geriatrics, Department of Medicine, Maccabi Healthcare Services, Tel Aviv, Israel; 17 Hadassah Medical Center and Faculty of Medicine, Hebrew University of Jerusalem, Jerusalem, Israel; 18 Amsterdam UMC Location University of Amsterdam, Internal Medicine, Section of Geriatric Medicine, Amsterdam, The Netherlands; 19 Amsterdam Public Health Research Institute, Amsterdam, The Netherlands; 20 Department of Clinical Pharmacology, Royal North Shore Hospital, Sydney, Australia; 21 Northern Clinical School, Faculty of Medicine and Health, University of Sydney, Sydney, Australia; 22 Division of Geriatric Medicine, UMass Chan Medical School, Worcester, MA USA

We note Dr. Wehling’s comments on our recent publication on potentially inappropriate prescribing cascades [1] (PIPCs) identified through a consensus Delphi process by an international expert panel. These PIPCs should alert clinicians when reviewing pharmacotherapy and making prescribing decisions. Our goal was to provide a resource to complement other available explicit prescribing tools (e.g., STOPP/START, FORTA, Beers Criteria) that can be employed by clinicians to optimize drug therapy. We contend that the current list of 65 explicit PIPCs represents a substantial addition to the literature.

The essence of a prescribing cascade is that the initial drug causes an adverse effect, which in turn leads to a subsequent drug being prescribed to manage this adverse effect. For PIPCs, this is typically due to misinterpreting the adverse effect as a new medical condition. As noted, prescribing cascades do not always stop with the development of one new condition, but the cascade of events can continue, often going unrecognized. As such, our list is an important starting point to encourage clinicians to reflect on their pharmacotherapy decisions and symptoms that may represent medication adverse effects rather than new clinical conditions.

While Dr. Wheling emphasizes multi-step prescribing sequences, evidence shows that even more simple prescribing cascades (i.e., initial drug to subsequent drug pairs), if unrecognized, frequently serve as the first step in the development of a longer prescribing cascade. Our list, therefore, also aids in being an early-warning framework in identifying prescribing cascades at their onset to prevent more complex inappropriate sequences from developing.

In our work with international leaders in pharmacotherapy for older adults from eight countries, a prime concern was to make sure that users of this list realized that all the prescribing cascades we identified through the Delphi process were only *potentially* inappropriate. We certainly agree that individual circumstances and clinical judgement need to guide all prescribing decisions. As such, the word “potentially” was included in the title and used throughout the text. We fully agree that in some circumstances, where benefits may outweigh risks, continuing the initial drug with appropriate management of its adverse effects is clinically indicated, and is also in the patient’s best interest. Our prescribing cascade resource is not meant to discourage such decisions, but to prompt prescribers to pause and consider deprescribing or dose adjustment as equally valid options.

We readily acknowledge that our PIPC list will never be complete. As pointed out, the number of these prescribing cascades is potentially enormous [2, 3]. Also, new prescribing cascades will be reported from drug therapies that are currently available and as new drug therapies come on the market; new prescribing cascades will undoubtedly be identified.

We agree that choosing an optimal initial drug is important, but doing this from the outset does not mean that prescribing cascades do not occur. While existing explicit prescribing tools provide valuable guidance on the appropriateness of individual medications, they do not explicitly flag potential prescribing cascades. Our PIPC list is unique in highlighting inter-drug dynamics where the adverse effect of one medication becomes the indication for another. As such, it complements, rather than duplicates, existing resources.

Our goal is to ensure that these 65 PIPCs are kept at the forefront of busy clinicians’ minds, when making medication-based decisions. Clinicians, together with their patients, should pause to consider the following three questions [4]:

- Is the new medication being prescribed to treat an adverse effect of a previous medication?
- Is the initial drug therapy still necessary, or could it be safely deprescribed or reduced in dosage?
- Are the benefits of continuing the initial medication and potentially the subsequent one, truly outweighing the risk?

Even if some prescribing cascades occur infrequently in highly optimized settings, when they do occur, they can contribute to morbidity, polypharmacy, and healthcare costs. Recognizing and addressing PIPCs early remains an important safeguard for patient safety, and therefore, having a list to highlight PIPCs is a valuable resource to prevent medication-related harm worldwide.
